# Editorial: Hospital acquired infection in patients with cardiovascular diseases

**DOI:** 10.3389/fmed.2026.1787585

**Published:** 2026-01-29

**Authors:** Siyi He, Jingwei Li

**Affiliations:** 1 Department of Cardiovascular Surgery, The General Hospital of Western Theater Command, Chengdu, Sichuan, China; 2 Department of Cardiology, Xinqiao Hospital, Army Medical University, Chongqing, China

**Keywords:** cardiovascular disease, hospital acquired infection, prevention, surveillance, treatment

Hospital acquired infection (HAI) refers to infections that occur during the treatment in a hospital, excluding infections that had already begun before admission or were already in the incubation period upon admission. Statistics released by the World Health Organization (WHO) reveal that approximately 8.9 million cases of HAIs occur annually in acute care settings and long-term healthcare institutions (1). In high-income countries, the prevalence of HAIs stands at 7.5%; however, separate research has cited rates ranging from 5.7% to 7.1% across European countries and 4.5% in the United States. In contrast, the prevalence of HAIs in low- and middle-income countries ranges between 5.7% and 19.2% (2). HAI not only significantly prolongs hospital stays, increases the risk of functional impairment and antibiotic resistance, but also potentially imposes a heavy burden on the healthcare system. With the intensification of population aging, the incidence of cardiovascular diseases (CVDs) has been rising year by year. Due to the weakened immune system and frequent invasive procedures, patients with CVDs has constituted a high-risk group for HAI (3). The dynamic changes in infection risks among different age groups and subpopulations make the prevention and control of HAIs even more challenging.

As the core process of HAI treatment, the pharmacotherapy for HAIs should follow the pathogen-oriented principle. Initially, empirical antimicrobial therapy should be implemented based on clinical characteristics and local bacterial resistance surveillance data. Subsequent adjustments are made to targeted therapy in conjunction with pathogen culture and drug sensitivity test results. Simultaneously, consideration should be given to the patient's underlying diseases. The meta-analysis completed by Peng et al. included 22 observational studies, systematically comparing the efficacy and safety of polymyxin B (PMB) with other antimicrobial agents in the treatment of HAIs, especially multidrug-resistant (MDR) Gram-negative bacterial infections. The results showed that compared with the control antimicrobial regimen, PMB did not reduce overall mortality, and when compared with ceftazidime-avibactam, the latter had a superior advantage in reducing mortality. In terms of safety, the incidence of nephrotoxicity with PMB was lower than that with colistin, but there was significant heterogeneity among the studies. Patients with CVDs often suffer from renal dysfunction and hemodynamic instability, necessitating a balance between “coverage of MDR pathogens” and “organ toxicity” when selecting antimicrobial agents. This study suggests that PMB is more suitable as a rescue treatment rather than a first-line regimen, and emphasizes that in intensive settings, priority should be given to novel β-lactamase inhibitor combinations to improve prognosis.

There is still controversy over whether covering catheters with antimicrobial agents can effectively prevent catheter-related nosocomial infections. The randomized controlled trial conducted by Dong et al. evaluated the effectiveness of polyhexamethylene biguanide (PHMB)-coated central venous catheters (CVCs) in reducing bacterial colonization at the catheter tip. The overall results showed that the PHMB coating did not significantly reduce the overall colonization rate, but certain benefits were observed in the male cancer patients undergoing abdominal surgery, along with a reduction in hospital stay. CVC is widely used in CCU, but this study demonstrates that antimicrobial coatings are not an effective solution. Infection prevention and control still rely on standardized catheter placement, maintenance procedures, and early catheter removal strategies. Further verification of its clinical value is needed in high-risk subgroups, such as patients with heart failure.

The prevalence of HAI varies significantly across different regions and institutions, which is closely linked to the accessibility and effectiveness of infection prevention and control strategies. Furthermore, the reported incidence of HAIs may be shaped by hospital-specific objectives or targets associated with quality improvement initiatives. The COVID-19 pandemic has not only posed severe challenges to HAI prevention and control, but also promoted the standardized construction and systematic improvement. Liao et al. compared the changes of HAI prevalence in the CCU of a tertiary general hospital in Beijing before and after the COVID-19 pandemic. The results showed that during the pandemic, the overall rate of HAI in the CCU decreased by approximately 20.6%. The study has convincingly demonstrated the significant effect of strictly implementing standardized measures on HAI prevention and control in CCU. For cardiovascular patients, HAI prevention and control does not solely rely on antimicrobial drugs, but rather depends more on systematic process management. In the future, the enhanced model during the pandemic should be normalized for application to patients with CVDs.

Strengthening the predictive ability of HAI, screening high-risk patients in advance, and implementing targeted interventions are also important means to prevent HAI. The study of Shen et al. included 675 elderly patients undergoing vascular surgery and found that preoperative frailty and moderate to severe malnutrition were independent risk factors for hospital-acquired pneumonia (HAP), with the area under ROC curve (AUC) for the combined prediction of HAP reaching 0.763. Among elderly cardiovascular patients, “frailty-malnutrition-infection” constitutes an important pathological chain. This study emphasizes the importance of incorporating comprehensive geriatric assessment into perioperative management of cardiovascular surgery. By means of preoperative nutritional intervention, respiratory function training, and other methods, the prevention and control of HAP can be moved forward.

Delicati et al. compared the oral and skin microbiome characteristics in patients undergoing vascular endografts (VEGs) implantation with and without HAIs through 16S rRNA sequencing. The results showed that the diversity of oral microbiota decreased in infected patients, while the diversity of skin microbiota increased. Potential infection risk markers, such as Firmicutes D, Staphylococcus, Haemophilus D, and Prevotella denticola were identified. This study extends the infection susceptibility from purely perioperative factors to the level of host microbiota, providing new insights for preoperative risk stratification in cardiovascular patients. Among high-risk patients undergoing cardiovascular surgery, combining microbiome characteristics with individualized infection prevention measures holds significant translational potential, such as oral management and skin colonization intervention.

Xi et al. reported a case of brachial arterial pseudoaneurysm caused by Listeria monocytogenes. The patient had a history of multiple vascular lesions and surgeries. After the pathogen was identified through blood culture, targeted anti-infective treatment was administered first, followed by surgical reconstruction, ultimately achieving a favorable prognosis. This case suggests that in patients with CVDs, atypical pathogens can also cause severe vascular infectious complications. For patients with recurrent vascular lesions, impaired immune function, or long-term hospitalization, vigilance should be heightened for rare pathogens such as Listeria, emphasizing the importance of blood culture and precise anti-infective treatment.

Current research on HAI in patients with cardiovascular diseases has shifted from passive treatment to active prediction and precise prevention. The focus of research has expanded from single pathogens and antimicrobial drug selection to host susceptibility, medical device-related risks, and optimization of systematic control processes. In the future, it will focus on precise risk stratification of HAI and achieve high-performance prediction by combining artificial intelligence technology. Strengthening equipment and environmental control technologies, and promoting a multidisciplinary collaborative management model, are also development trends in HAI prevention and control. The aforementioned approach holds promise for systematically reducing the incidence of HAI in patients with cardiovascular diseases and improving their long-term prognosis, without increasing antibiotic pressure.

## References

[1] Guidelines on Core Components of Infection Prevention and Control Programmes at the National and Acute Health Care Facility Level. Geneva: World Health Organization (2016). 27977095

[2] Allegranzi B Bagheri Nejad S Combescure C Graafmans W Attar H Donaldson L . Burden of endemic health-care-associated infection in developing countries: systematic review and meta-analysis. Lancet. (2011) 377:228–41. doi: 10.1016/S0140-6736(10)61458-4 21146207

[3] Vîrtosu DM Munteanu Dragomir A Crişan S Luca S Pătru O Băghină RM . Prevalence of healthcare-associated infections in patients with cardiovascular diseases: a literature review. J Clin Med. (2025). 14:4941. doi: 10.3390/jcm14144941 40725634 PMC12294981

